# Open Source Tools and Methods: A New Category of Short Papers to Share Knowledge, Accelerate Research, and Acknowledge Those Who Develop Such Tools and Methods

**DOI:** 10.1523/ENEURO.0342-19.2019

**Published:** 2019-09-05

**Authors:** Christophe Bernard

**Affiliations:** Editor-in-Chief

I am excited to introduce a new type of paper at eNeuro to provide a venue to share open-source tools and methods.

Neuroscience relies heavily on the development of new tools to perform research and methods to acquire and analyze data. For example, there is a considerable amount of developed knowledge in terms of tools and methods behind fMRI. With the acquisition of very large datasets, such as thousands of cells simultaneously recorded with optical or electrophysiological equipment, laboratories must develop specific tools and methods, often in-house, to acquire and analyze data. We can anticipate the development of tools to extract relevant information from multidimensional datasets, big data, etc. For such types of papers, we already have the Methods/New Tools submission type. But the development of tools and methods is not limited to major pieces of equipment, large software, etc. We will always need a low-cost device to measure animal behavior, a new biophysical model of a single neuron, a better method to realign images when performing *in vivo* two-photon imaging, scripts and codes to analyze signals, etc. Such tools and methods often end up being published in the Materials and Methods section of a paper. However, such tools and methods may be very useful to the community if they could be made available to all of us in a plug-and-play manner. See the recent commentary by White et al. (2019) for more information on the benefits of open source in neuroscience. And as there is no publication venue dedicated explicitly to Open Source Tools and Methods in neuroscience, we decided to create one at *eNeuro*.

The requirements are relatively simple. The papers should be short and fully describe the tool or the method so that other scientists can use them without having to contact the authors for details.

Introducing this new type of papers offers several advantages: reducing the time and money laboratories spend to reinvent methods; increasing transparency; and improving reproducibility. As importantly, it allows for acknowledgment of those who developed such tools and methods fully, often rotating students or engineers recruited on a short-duration contract. On a standard research paper, their name ends up in the middle of the list of authors, but the Open Source Tools and Methods type will allow them to be the first author.

I am sure that we all have such tools and methods stored away in our drawers, which we would like to be published and used by others (I do). This new initiative of *eNeuro* is for you.

*eNeuro*: always leading.
